# Signaling a link between interferon and the traits of Down syndrome

**DOI:** 10.7554/eLife.20196

**Published:** 2016-09-06

**Authors:** Gina Kirsammer, John D Crispino

**Affiliations:** Department of Medicine, Northwestern University, Chicago, United States; Department of Medicine, Northwestern University, Chicago, United Statesj-crispino@northwestern.edu

**Keywords:** down syndrome, trisomy 21, interferon, JAK inhibitors, ruxolitinib, Human, Mouse

## Abstract

Elevated interferon signaling is a hallmark of Down syndrome.

**Related research article** Sullivan KD, Lewis HC, Hill AA, Pandey A, Jackson LP, Cabral JM, Smith KP, Liggett LA, Gomez EB, Galbraith MD, DeGregori J, Espinosa JM. 2016. Trisomy 21 consistently activates the interferon response. *eLife* **5**:e16220. doi: 10.7554/eLife.16220

Most people have two copies of every chromosome in their cells. However, Down syndrome, one of the most complex human genetic disorders, is caused by the presence of a third copy of chromosome 21 in some or all of an individual’s cells. Also known as trisomy 21, Down syndrome occurs in approximately 1 in 700 births in the United States. Traits that are commonly seen in individuals with Down syndrome include intellectual disability, heart defects, Alzheimer’s disease, susceptibility to leukemia, and a decreased likelihood of developing other tumors (Alexander et al., 2016; Hasle et al., 2016).

Despite much research, the genetic mechanisms that link trisomy 21 to specific Down syndrome traits are poorly understood, in part due to the large number of genes involved. Additionally, genetic modifiers affect how common and severe the traits that result from trisomy 21 will be in different individuals. This complexity has made it difficult to identify the molecules that could be therapeutically targeted to improve the quality of life for individuals with Down syndrome.

Now, in eLife, Joaquín Espinosa and colleagues at the University of Colorado – including Kelly Sullivan as first author – shed new light on the link between the multiple traits associated with Down syndrome and the extra chromosome 21 genes inside cells affected by trisomy 21 (Sullivan et al., 2016). Their results suggest that interferons – signaling molecules that are normally released by cells in response to nearby pathogens – may underlie many of the features associated with Down syndrome.

By sequencing the RNA of human fibroblast cells taken from age- and gender-matched individuals with and without Down syndrome, Sullivan et al. identified a consistent core pattern of gene expression in cells affected by trisomy 21. As expected, these cells contained approximately 1.5 times as much of the gene products for chromosome 21 genes as normal cells. Unexpectedly though, Sullivan et al. also found that the core gene expression pattern associated with trisomy 21 was associated with an interferon-stimulated transcriptional response. It is worth noting that four of the six interferon receptors are encoded on chromosome 21, which partially explains why the interferon pathway is activated.

Next, Sullivan et al. performed several studies to verify the relevance of the interferon pathway to Down syndrome traits. First, they showed that interferon stimulation produced a stronger response in Down syndrome fibroblasts than in control cells. Second, using an shRNA screen of the protein kinases encoded in the genome of the fibroblasts, they identified two interferon-activated kinases, which strongly reduce the viability of Down syndrome fibroblasts (Figure 1). Treating the trisomy 21 cells with a drug that inhibits the kinases recovered their viability.

**Figure 1.**
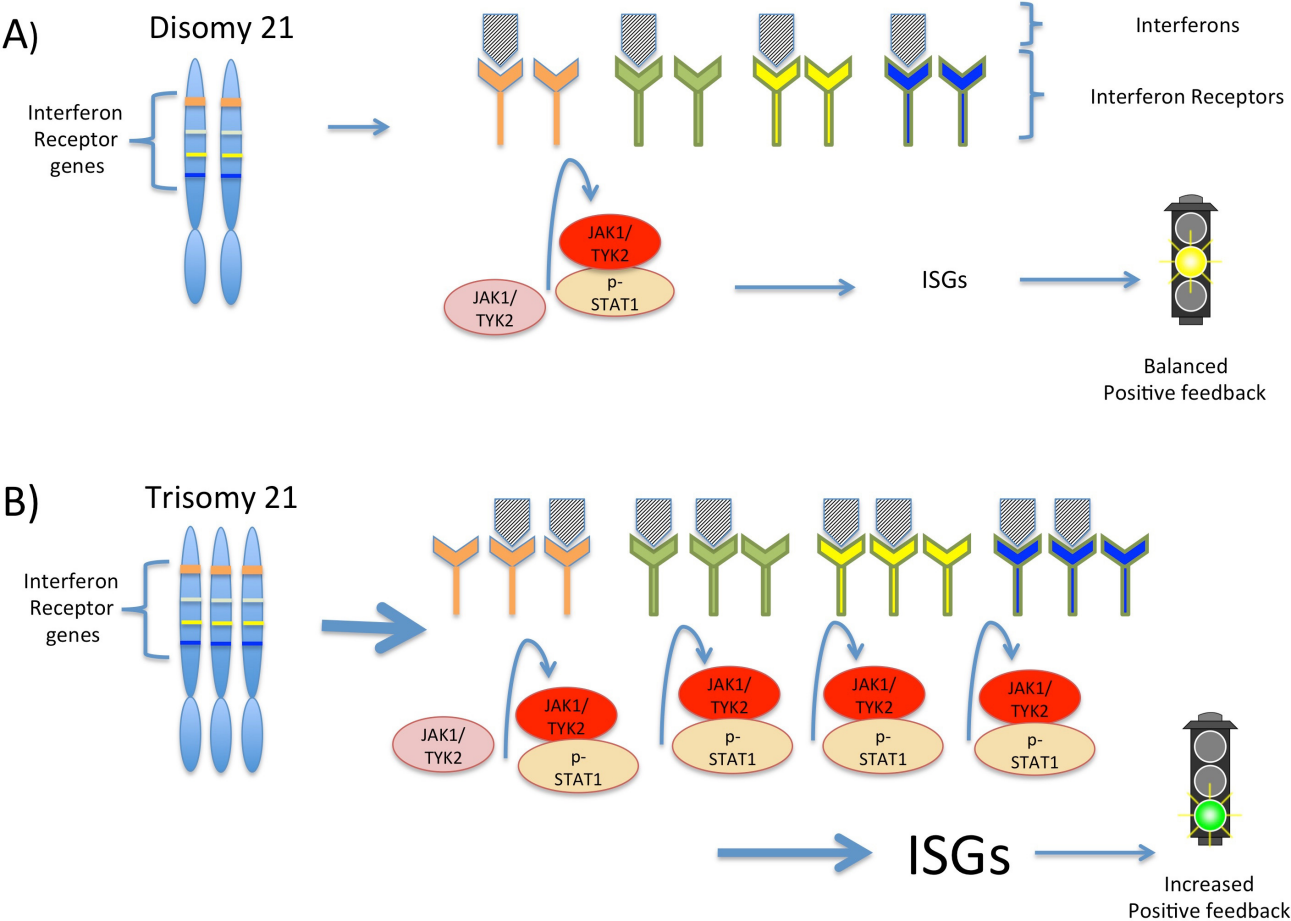
Interferon signaling is amplified by trisomy 21. (**A**) Chromosome 21 encodes four types of interferon receptors. In cells with two copies of this chromosome (disomy 21), the binding of interferons to the receptors activates two kinases, JAK1and TYK2. These kinases activate the transcription factor STAT1, which then transcribes a set of genes known as interferon stimulated genes (ISGs). (**B**) In cells with three copies of chromosome 21 (trisomy 21), as in Down syndrome, the increased abundance of interferon receptors increases the transcription of ISGs, including the genes that encode interferons themselves. This ultimately leads to increased positive feedback and hyperactivated interferon signaling, which can damage the cell.

Sullivan et al. next confirmed their findings in multiple types of blood cells from individuals with and without Down syndrome. While gene expression analysis showed that all cells with trisomy 21 had activated an interferon response, the specific genes stimulated by interferon signaling varied by cell type. Sullivan et al. then examined gene expression in a mouse model of trisomy 21 (Li et al., 2007) and found interferon activation in multiple blood cell types.

By distinguishing between chromosome 21 genes and non-chromosome 21 genes in their data, Sullivan et al. have developed a model that suggests how the extra copy of chromosome 21 produces the traits of Down syndrome. In the model, the increased expression of the four interferon receptors encoded on chromosome 21 increases interferon signaling in Down syndrome cells, leading to positive feedback and greater amplification of interferon signaling than would be expected from the increased number of gene copies alone (Figure 1). This increased signaling, in turn, increases the expression of different interferon response genes in different cell types, which potentially explains the variability observed in the traits associated with trisomy 21.

While interferon sensitivity in Down syndrome has previously been reported (Tan et al., 1974), the study by Sullivan et al. establishes a broad foundation of RNA, protein and functional data that provide insights into how interferon signaling contributes to the distinct characteristics of Down syndrome. Furthermore, the study highlights the potential of targeting interferons as a way of treating these symptoms.

Intriguingly, a number of Down syndrome traits resemble the symptoms of hyperactive interferon signaling disorders, termed “interferonopathies” (Crow and Manel, 2015). In a mouse model of Down syndrome, both anti-interferon therapy and genetic methods that reduce the number of interferon receptors on the surface of cells have been shown to improve growth and brain development (Maroun et al., 2000). Moreover, the drug memantine, which can improve cognition in individuals with Down syndrome (Costa, 2014), works by blocking NMDA receptor signaling. Interferons are known to amplify signaling via NMDA receptors (Sonekatsu et al., 2016), which correlates with amplified interferon signaling in Down syndrome and may suggest a strategy for combined therapy.

The next step will be to determine whether specific targets of interferon signaling can be linked to Down syndrome traits. It also remains to be discovered whether interferon therapy might be useful to individuals with Down syndrome – either alone, or in combination with other treatments – and whether there are developmentally sensitive time-points for potential intervention.

Finally, interferon signaling has well-established functions in regulating tumor development. Individuals with Down syndrome experience a unique cancer risk profile: although they have a dramatically increased risk of developing childhood leukemia, they are far less likely to develop solid tumors than individuals with just two copies of chromosome 21 (Hasle et al., 2016). It remains to be seen whether the cell-type specific effects of interferon signal amplification underlie these differences in tumor susceptibility. In any case, insights into the correlation between hyperactive interferon signaling in Down syndrome and cancer development may provide opportunities for treating both Down syndrome-associated malignancies and cancer in the general population.
